# Field and Laboratory Studies on Pathological and Biochemical Characterization of Microcystin-Induced Liver and Kidney Damage in the Phytoplanktivorous Bighead Carp

**DOI:** 10.1100/tsw.2008.32

**Published:** 2008-02-06

**Authors:** Li Li, Ping Xie, Longgen Guo, Zhixin Ke, Qiong Zhou, Yaqin Liu, Tong Qiu

**Affiliations:** ^1^ Donghu Experimental Station of Lake Ecosystems, State Key Laboratory for Freshwater Ecology and Biotechnology of China, Institute of Hydrobiology, The Chinese Academy of Sciences, Wuhan 430072, China; ^2^ Fisheries College of Huazhong Agricultural University, Wuhan, 430070, China

**Keywords:** phytoplanktivorous bighead carp, microcystin, liver, kidney, pathological and biochemical characterizations, large fish pen

## Abstract

Field and experimental studies were conducted to investigate pathological characterizations and biochemical responses in the liver and kidney of the phytoplanktivorous bighead carp after intraperitoneal (i.p.) administration of microcystins (MCs) and exposure to natural cyanobacterial blooms in Meiliang Bay, Lake Taihu. Bighead carp in field and laboratory studies showed a progressive recovery of structure and function in terms of histological, cellular, and biochemical features. In laboratory study, when fish were i.p. injected with extracted MCs at the doses of 200 and 500 μg MC-LReq/kg body weight, respectively, liver pathology in bighead carp was observed in a time dose-dependent manner within 24 h postinjection and characterized by disruption of liver structure, condensed cytoplasm, and the appearance of massive hepatocytes with karyopyknosis, karyorrhexis, and karyolysis. In comparison with previous studies on other fish, bighead carp in field study endured higher MC doses and longer-term exposure, but displayed less damage in the liver and kidney. Ultrastructural examination in the liver revealed the presence of lysosome proliferation, suggesting that bighead carp might eliminate or lessen cell damage caused by MCs through lysosome activation. Biochemically, sensitive responses in the antioxidant enzymes and higher basal glutathione concentrations might be responsible for their powerful resistance to MCs, suggesting that bighead carp can be used as biomanipulation fish to counteract cyanotoxin contamination.

### INTRODUCTION

Cyanobacteria, specifically *Microcystis aeruginosa*, pose a threat to animal and human health due to their ability to produce the hepatotoxic heptapeptides, microcystins (MCs)[1,2]. Among more than 70 MC isoforms, MC-LR is the most common variant, followed by MC-RR and MC-YR[3]. MCs are taken up by hepatocytes via multispecific bile acid transporters, and can potently inhibit serine/threonine protein phosphatases 1 and 2A[4], inducing hyperphosphorylation of cellular proteins and disruption of the hepatocyte cytoskeleton due to disorganization of cytoplasmic microtubules, cytokeratin intermediate filaments, and actin microfilaments[5,6]. Thus, acute MC poisoning in mammals is histologically characterized by rounding of the hepatocytes and disruption of the liver structure, which induces intrahepatic hemorrhage and leads to death[4]. Up to now, different exposure routes have been used to evaluate the impact of MCs on fish, including intraperitoneal (i.p.) injection[7,8,9,10], oral gavaging via the diet[8,11,12,13], and immersion in water containing purified MCs or lysates or whole cells of cyanobacteria[8]. However, these studies mainly focused on carnivorous rainbow trout[9,11] and omnivorous common carp[8,12,13] and golden fish[10], while there is only limited information on phytoplanktivorous fish that are more frequently exposed to cyanobacterial toxins in natural conditions because of habitat and feeding mode.

Recent studies suggest that oxidative stress plays an important role in the pathogenesis of MC toxicity. Previous studies demonstrated that cyanobacterial extracts and pure MCs induce oxidative stress through increasing reactive oxygen species (ROS) and oxidative damage products, such as lipid peroxides in many organisms including fish[6,14,15,16,17]. Both ROS and lipid peroxides are known to be reduced (scavenged) by nonenzymatic defense chemicals, such as vitamins E, C, A, and glutathione (GSH), and specific antioxidant enzymes, such as catalase (CAT), superoxide dismutase (SOD), glutathione peroxidase (GPX), and glutathione S-transferase (GST)[18]. To the extent of our knowledge, effects of MCs on these antioxidant defense systems of fish have only been measured in *Danio rerio* embryos[19,20], in isolated hepatocytes of common carp[6], and in the livers of juvenile goldfish (*Carassius auratus* L.) and tilapia fish (*Oreochromis* sp.) either injected i.p. with MC-LR or fed with toxic cyanobacterial cells[16,21]. However, such studies are still lacking from fish in field experiments.

Herbivorous fish are among the most abundant fish groups. The phytoplanktivorous fish are especially important to humans because of their roles in aquatic ecosystems as direct consumers of phytoplankton primary production, their potential for biological management of cyanobacterial blooms, and their values as food fish[22,23]. Bighead carp, *Aristichthys nobilis* (Richardson), is one of the most important phytoplanktivorous fish in China, and has been introduced worldwide for aquaculture, comprising as much as 6% of the total freshwater fish production of the world[24]. Bighead carp was also suggested to be able to suppress and graze out *M. aeruginosa* blooms[23]. Feeding habits (carnivorous/herbivorous) lead to different uptake of MCs, mainly by the diet itself, but also by the longer, hence more thorough, digestion of the herbivorous fish[12]. Preliminary studies in our laboratory suggested that phytoplanktivorous silver carp are capable of degrading MC-LR actively and inhibiting its transportation across the intestines[25]. A better understanding of the harmful effects of MCs on fish is necessary to improve our knowledge on toxication and antioxidation mechanisms involved in the toxicity of MCs, especially as fish of different trophic levels react in different ways, or with different intensities, to the presence of MCs.

This paper aims to investigate histopathological characterizations and biochemical responses in the antioxidant defense system, including CAT, SOD, GPX, GST, and GSH in the liver and kidney of bighead carp after i.p. administration of MCs under laboratory conditions and exposure to natural cyanobacterial blooms in Meiliang Bay of Taihu, in order to further understand the hepatic toxication and antioxidation mechanism in fish.

## MATERIALS AND METHODS

### Laboratory Study

The freeze-dried cyanobacterial materials used in the experiment were collected from Lake Dianchi, Yunnan of China. MCs were prepared and measured as previously described[26] with some improvements. Briefly, the materials were extracted three times with 10 ml of 75% methanol (v/v) for 3 h at 4°. The extract was centrifuged at 10,000 *g* for 30 min, and the supernatants were pooled and applied to a $C_{18}$ cartridge (Dalian Institute of Chemical and Physical, China). The cartridge containing MCs was rinsed with 10 ml water and MCs were finally eluted from the $C_{18}$ cartridge with 10 ml of methanol. The eluate was evaporated under reduced pressure and then the residue was dissolved in water. After a reverse-phase high-performance liquid chromatography (HPLC) (Shimadzu, LC-10AD) analysis, the MC content in the cyanobacterial material was 1.41 mg/g dry weight (DW), among which MC-RR, -LR, and -YR were 0.84, 0.50, and 0.07 mg/g DW, respectively.

Juvenile fish of bighead carp with a mean weight of 51 ± 2 g were purchased from a local fish hatchery (Wuhan, China). Fish were acclimated for 3 days prior to experimentation in 100-l aquaria containing dechlorinated tap water. Water temperature was 20 ± 1°C, pH maintained 7.4, and dissolved oxygen values were 7.5 mg/l. No food was given to the fish throughout the experiment. In this experiment, fish were randomly divided into three groups: fish in the low-dose group (n = 15) and high-dose group (n = 15) were injected i.p. with crude extracted MCs (mainly MC-RR and -LR) at 200 and 500 μg MC-LReq/kg body weight (bw), respectively; the control fish (n = 6) were injected i.p. with an equal volume of distilled water. In this study, coefficients of 0.2 and 0.4 were used to convert MC-RR and -YR into the MC-LR equivalent, respectively, since i.p. $LD_{50}$ in mice for MC-RR and -YR are about 5 and 2.5 times higher than that for MC-LR, respectively[27]. Three MC-treated fish in both dose groups were killed at 1, 3, 12, 24, and 48 h postinjection, respectively. Three control fish for each time were killed at 0 and 48 h. Livers were excised, freed of attached tissue, and divided into two parts: one was immediately frozen in liquid nitrogen before storage at –80°C for biochemical analysis and the other was fixed for microscopic examination.

### Field Study

Lake Taihu, the third largest freshwater lake in China, is located in Jiangsu province, eastern China. Meiliang Bay (water surface area 135 $km^2$), a part of Taihu Lake, accommodates municipal and industrial wastewater from Wuxi City, but also acts as the principal water source for the city. Meiliang Bay is the most eutrophic part of the lake, characteristic of extremely dense accumulation of toxic *Microcystis* blooms by wind in the summer. A large fish pen was built in Meiliang Bay in 2003 (120°12′46′′~120°13′07′′E, 31°29′07′′~31°29′55′′N) (Fig. 1), with a total area of 1.08 $km^2$ and a mesh size of 2 × 2 cm. Bighead carp with a mean weight of 264 ± 24 g were bought from a local fishery and put into the pen at the beginning of March 2004. Totally, approximately 6500-kg bighead carp fingerlings were stocked. During the first couple of weeks, dead fish were occasionally found due to injury of transportation of the fingerlings. Afterwards, no visible fish death was found in the fish pen. From April 2004 to March 2005 (12-month period), five bighead carp in the pen were captured monthly using gill nets, and then measured, weighed, and sacrificed immediately. Samples of liver and kidney were excised and divided into two parts: one was immediately frozen in liquid nitrogen before storage at –80°C for biochemical analysis and the other was fixed for microscopic examination (samples for electron microscopic examination were merely prepared in July and December). Foregut contents were also collected from the proximate end of the intestine to the middle of the first loop, and preserved with Lugol's iodine solution immediately for examining food items. Water temperature was recorded by a WMY-01 digital thermometer in the field. Dissolved oxygen and pH were determined with an Orion 810 dissolved oxygen meter and PHB-4PH meter, respectively.

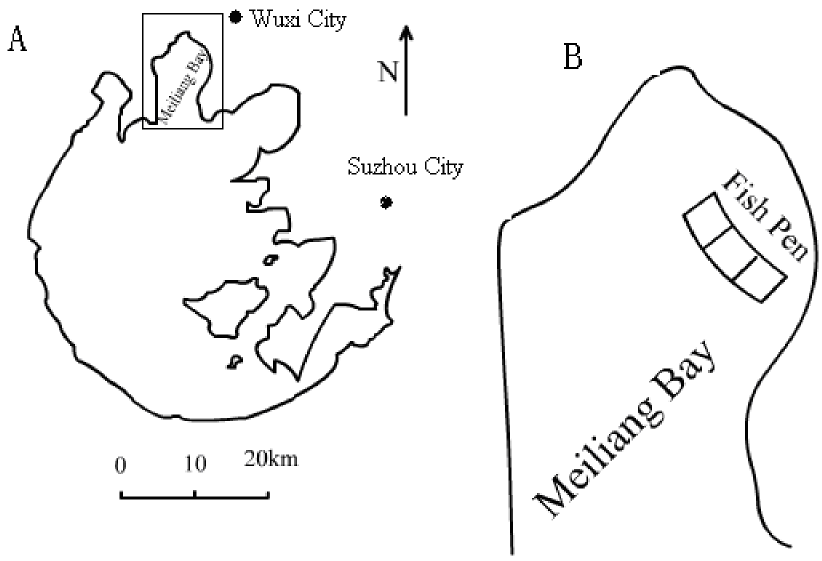

**FIGURE 1.** Sketch map of Taihu Lake (A) and the location of the fish pen in Meiliang Bay (B).

For phytoplankton estimation, we set two sampling sites in the fish pen. At each site, water samples were collected monthly from the surface and near the bottom using a 5-l modified Patalas's bottle sampler, and a 1-l mixed water sample was preserved with 1% acidified Lugol's iodine solution and concentrated to 30 ml after sedimentation for 48 h. After mixing, 0.1-ml concentrated sample or gut content sample were counted directly under 400× magnification. Colonial *Microcystis* cells were separated using a high-speed blender (Ultra-Turrax) and counted as above. Taxonomic identification was made according to Hu et al.[28] and biomass was estimated from approximate geometric volumes of each taxon, assuming that 1 $mm^3$ equals $10^{-6}$ µg fresh weight.

### Histopathology

For light microscopic study, samples were first fixed in Bouin's fixative for 24 h and then immediately dehydrated in graded series of ethanol, immersed in xylol, and embedded in paraffin wax. Sections of 4 µm were mounted. After they had been deparaffinized, the sections were rehydrated, then stained with hematoxylin and eosin (H&E). Histopathological assessment was done on a light microscope at 400× magnification.

For transmission electron microscopic study, samples were prefixed in 2.5% glutaraldehyde solution, diced into 1 $mm^3$, followed by three 15-min rinses with 0.1*M* phosphate buffer (pH 7.4). Postfixation was in cold 1% aqueous osmium tetroxide for 1 h. After rinsing with phosphate buffer again, the specimens were dehydrated in a graded ethanol series of 50–100% and then embedded in Epon 812. Ultrathin sections were sliced with glass knives on a LKB-V ultramicrotome (Nova, Sweden), stained with uranyl acetate and lead citrate, and examined under a HITACHI, H-600 electron microscope.

### Biochemical Analyses

Tissue samples were homogenized (1:10, w/v) in a cold (4°C) buffer solution containing Tris base (20 m*M*), EDTA (1 m*M*), dithiothreitol (1 m*M*; Sigma), sucrose (0.5 m*M*), KCl (150 m*M*), and phenylmethylsulfonyl fluoride (1 m*M*; Sigma), with pH adjusted to 7.6. Homogenates were centrifuged at 9500 *g* (4°) for 20 min and the supernatants used as enzyme source. CAT activity was determined spectrophotometrically by measuring the disappearance rate of hydrogen peroxide ($H_2O_2$) at 240 nm according to the method of Aebi[29] and expressed as µmoles $H_2O_2$/min/mg protein. SOD activity assay was based on the method described by Bayer and Fridovich[30]. One SOD unit was defined as 50% inhibition of the nitroblue tetrazolium (NBT) photoreduction to blue formazan and expressed as specific activity (units/mg protein). GPX activity was measured as decribed by Drotar et al.[31], using GSH as substrate, and expressed in nmoles/min/mg protein. GST activity was detected using the method of Habig et al.[32] by evaluating the conjugation of GSH (1 m*M*, Sigma) with the standard model substrate 1-chloro-2,4-dinitrobenzene (CDNB) (1 m*M*, Sigma) and expressed in nmoles/min/mg protein. GSH concentration was measured by the method of Griffith[33] and expressed as µg/mg protein. Protein contents were determined by the Coomassie blue method[34] using bovine serum albumin as a standard. All the experiments were carried out in triplicate.

### Statistical Analyses

Statistical analysis was undertaken using SPSS 11.5 for Windows. Values were expressed as mean ± standard deviation (SD). Data of biochemical parameters were subjected to analysis of two-way ANOVA followed by a posthoc multiple comparisons test (Bonferroni's test). Normality and variance homogeneity were previously verified. Differences were considered to be significant at level $p < 0.05$.

## RESULTS

### Laboratory Study

Control bighead carp presented with normal histology (Fig. 2A). In both dose groups, disassociation of some hepatocytes was observed as early as 1 h postinjection. In the 500-µg MC-LReq/kg dose group, changes appeared primarily in the pericentral region of the liver and were characterized by the appearance of hepatocytes with condensed cytoplasm 1 h after injection. Between 3 and 12 h, the typical chord structure disappeared, and condensation and vacuolation in the cytoplasm of the hepatocytes became more conspicuous and extended to a larger area of the liver (Fig. 2B). In addition, slight hemorrhaging via a ruptured vessel was observed. After 24 h, hemorrhage and tissue damage extended to the whole liver. Pyknotic nuclei (typical apoptotic nuclei) were noticed in the 200-µg MC-LReq/kg dose group (Fig. 2C). In the 500-µg MC-LReq/kg dose group, most of the cells were swollen and had lost their granular appearance. Additionally, their nuclei were faint or not visible, and were most likely necrotic (Fig. 2E). After 48 h, the initial tissue structure was reconstituted in both dose groups (Fig. 2D) and only few inflammatory regions persisted in the 500-µg MC-LReq/kg dose group (Fig. 2F). Moreover, the recovery of the tissue structure and regeneration of hepatocytes were obviously less visible in the 500-µg MC-LReq/kg dose group than in the 200-µg MC-LReq/kg dose group (Figs. 2D and F). Apparently, alterations in the organization of hepatocytes after i.p. injection of MCs were more rapid and severe in the high-dose group than in the low-dose group.

The activities of antioxidant enzymes CAT, SOD, GPX, and GST and GSH contents in bighead carp liver in both dose groups are presented in Table 1. There was no significant difference in all these parameters between the two dose groups throughout the experiment. CAT activity in the liver of bighead carp in both dose groups increased significantly ($p < 0.05$) at 3 h postinjection compared with the control. Similarly, there was also a significant induction of SOD activity ($p < 0.05$) at 3 h in both dose groups when compared to the control. GPX activity in the 500-µg MC-LReq/kg dose group was significantly higher ($p < 0.05$) at 3 h than in the control, but such difference was not significant ($p > 0.05$) in the 200-µg MC-LReq/kg dose group. For GST, significant increases were observed at 3 h postinjection ($p < 0.05$) in both dose groups, followed by complete recovery at 48 h. GSH levels of both dose groups remained rather stable over the 48-h experimental period and differed little from that of the control group.

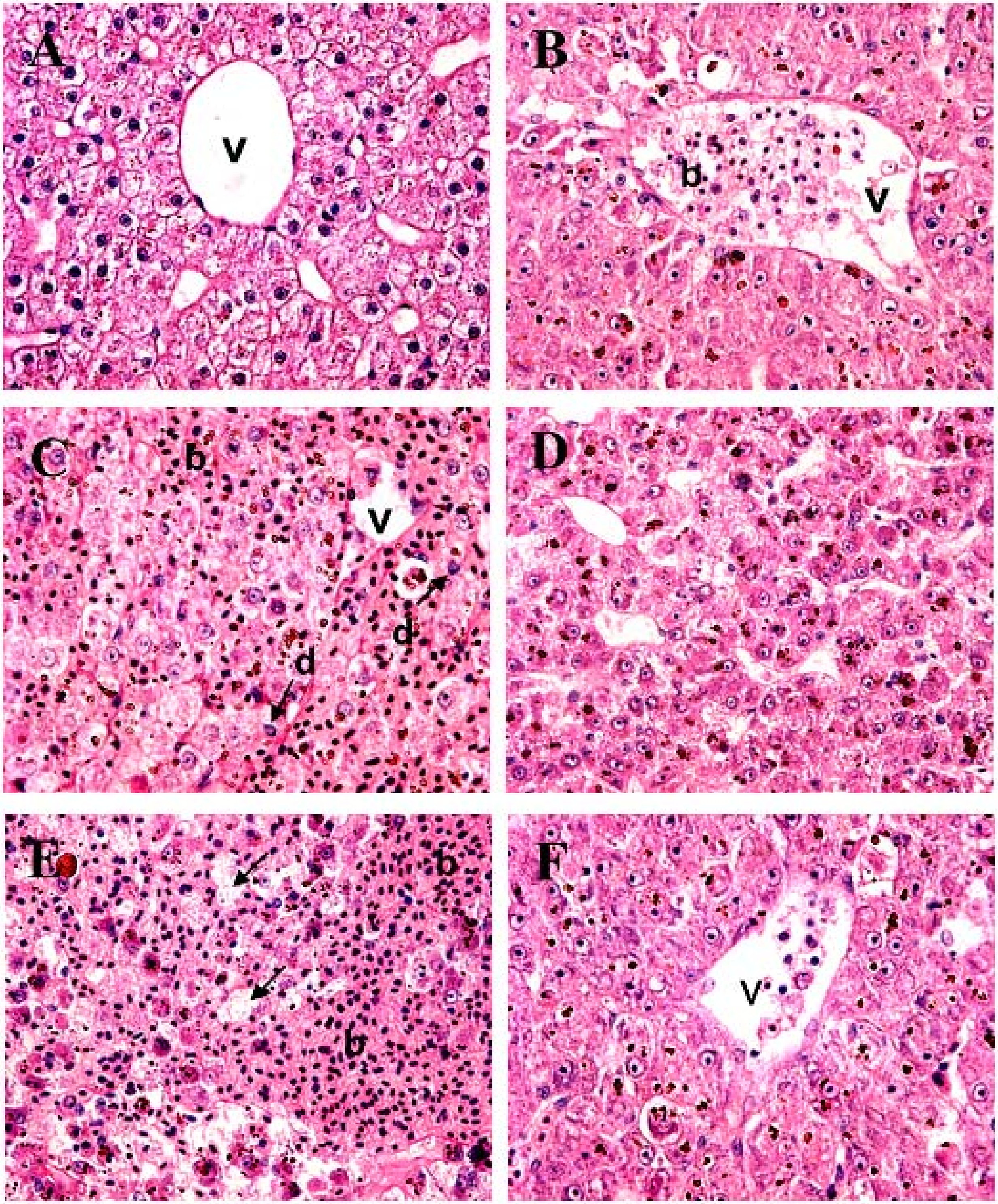

**FIGURE 2**. H&E-stained sections of livers from bighead carp injected i.p. with 200 and 500 µg MC-LReq/kg bw, respectively (400× magnification). (A) Control bighead carp; (B–D) bighead carp in 200-µg MC-LReq/kg dose group; (B) 3 h postinjection: rounding and vacuolation of pericentral hepatocytes; (C) 24 h postinjection: hemorrhage and highly condensed chromatin; (D) 48 h postinjection: great recovery of the initial tissue structure and regeneration of hepatocytes; (E and F) bighead carp in 500-µg MC-LReq/kg dose group; (E) 24 h postinjection: extensive hemorrhage and tissue damage involved the whole liver with loss of nuclei and lysis of membranes (arrows); (F) 48 h postinjection: obvious recovery of hepatocytes. b, blood cells; d, condensed chromatin, typical of apoptosis; v, central vein.

**TABLE 1**
**The Activities of Antioxidant Enzymes CAT, SOD, GPX, and GST and GSH Levels in the Liver of Bighead Carp Injected i.p. with 200 and 500 μg MC-LReq/kg bw, Respectively**

| Groups | Time (h) | Antioxidant enzymes in liver CAT | SOD | GPX | GST | GSH |
|---|---|---|---|---|---|---|
| Control | 0(48) | 285.8±32.0 | 11.3±1.8 | 144.4±6.0 | 95.3±7.2 | 57.8±5.2 |
| 200 μg/kg | 1 | 322.5±6.7 | 16.0±1.1 | 153.3±23.9 | 131.7±33.6 | 61.4±7.5 |
| | 3 | **453.1±24.2*** | **19.0±2.8*** | 187.9±40.5 | **164.5±20.5*** | 59.56±7.2 |
| | 12 | 337.2±37.8 | 16.6±1.2 | 159.3±17.3 | 77.1±18.5 | 58.5±3.5 |
| | 24 | 293.5±10.2 | 12.7±2.2 | 126.4±34.1 | 73.8±8.6 | 53.4±2.1 |
| | 48 | 273.9±72.7 | 16.6±1.0 | 122.6±39.4 | 104.0±1.0 | 66.4±2.1 |
| 400 μg/kg | 1 | 236.9±36.8 | 17.0±2.8 | 174.4±36.3 | 88.4±17.7 | 61.2±8.8 |
| | 3 | **450.07±41.2*** | **22.2±1.0**** | **211.3±19.0*** | **163.4±14.0**** | 51.8±1.8 |
| | 12 | 215.8±64.1 | 15.6±2.0 | 181.5±29.0 | 101.8±17.8 | 59.4±3.4 |
| | 24 | 164.2±50.6 | 11.4±0.5 | 133.9±5.6 | 93.3±4.7 | 55.2±1.3 |
| | 48 | 185.5±35.7 | 17.2±1.5 | 137.8±15.2 | 90.8±0.7 | 63.0±7.7 |
| ANOVA Effects | | (D) P<0.01 | (D) N.S | (D) N.S | (D) N.S | (D) N.S |
| | | (T) P<0.001 | (T)P<0.001 | (T) P<0.01 | (T) P<0.001 | (T) N.S |
| | | D*T P<0.05 | D*T N.S. | D*T N.S. | D*T P<0.05 | D*T N.S. |

*Note:* Results are expressed as mean ± SD (n = 3). As we did not find significant difference in the major biochemical parameters between 0 and 48 h of the control fish using an unpaired two-tailed Student's t-test ($p > 0.05$), we took the mean values of 0 and 48 h as controls. The statistical significance was analyzed by two-way ANOVA with doses (D) and time (T) as factors. Posthoc comparison for the effect of exposure time: Bonferroni's test, *$p < 0.05$, **$p < 0.01$.

### Field Study

According to occurrence and disappearance of *Microcystis* blooms in the fish pen, we divided the study period into three phases: before, during, and after *Microcystis* blooms (Table 2). The biomass of total phytoplankton, cyanobacteria, and *Microcystis* in the fish pen during *Microcystis* blooms was obviously higher than those before and after *Microcystis* blooms. During July-October, 52~89% of the total algal biomass was from *M. aeruginosa*; water temperature increased progressively, while dissolved oxygen content decreased slightly during *Microcystis* blooms. During the whole study period, pH maintained stable. Meanwhile, our microscopic examinations found that the major phytoplankton species in the gut content of bighead carp were similar with those in the lake water, and in July and August, almost 100% of the phytoplankton in the gut contents of bighead carp were from *M. aeruginosa*, indicating massive ingestion of *Microcystis.*

The livers of bighead carp collected from the fish pen showed a normal histological appearance during April and May (Fig. 3A). In June, alterations in the structural organization of hepatocytes were observed, which appeared to be initially restricted to the portal regions of the liver. During July and August, pathology was characterized by a loss of adherence between hepatocytes, a disorganization of typical chord liver

**TABLE 2**
**The Mean Values of Physical Parameters and the Phytoplankton Biomass in the Fish Pen Before, During, and After *Microcystis* Blooms**

| Parameters | Before *Microcystis* blooms (2004.4-2004.5) | During *Microcystis* blooms (2004.6-2004.10) | After *Microcystis* blooms (2004.11-2005.3) |
|---|---|---|---|
| Total phytoplankton biomass (mg/L) | 7.44±2.46 | 21.90±20.87 | 2.90±1.84 |
| Cyanobacterial biomass (mg/L) | 0.18±0.23 | 17.62±19.81 | 0.26±0.40 |
| *Microcystis* biomass (mg/L) | 0 | 16.99±19.21 | 0.21±0.40 |
| Temperature (? ) | 21.2±4.36 | 26.87±6.56 | 8.57±4.47 |
| Dissolved oxygen (mg/L) | 8.09±0.69 | 7.67±0.80 | 9.04±2.59 |
| pH | 8.01±0.62 | 8.77±0.95 | 8.17±0.17 |

structure and cytoplasmic vacuolation, particularly in pericentral regions (Figs. 3B and C). From September to October, recovery of liver structure and regeneration of hepatocytes were visible. From November 2004 to March 2005, the initial tissue structure was reconstituted and the histology of the liver was normal (Fig. 3D). Under transmission microscope, the liver of bighead carp showed numerous various-sized lipid droplets, vacuoles with myelin bodies, and the increase and proliferation of lysosomes in cytoplasms in July (Figs. 4A and B), but in December, we observed recovery of hepatocytes with abundant mitochondria and stacks of endoplasmic reticulum (Figs. 4C and D).

During April and May, histology of a healthy kidney was observed in bighead carp collected from the fish pen (Fig. 5A). Companied with the liver damage, histopathologic changes in the kidney were present in June, but were the most serious during July and August. Changes consisted of a dilation of the Bowman's capsule in the glomeruli and generalized vacuolation in the proximal tubular lining (Fig. 5B). However, the kidney structure of bighead carp recovered progressively during September and October. From November 2004 to March 2005, the kidney of bighead carp recovered completely (Fig. 5C). Ultrastructural changes in the kidney in July were characterized by a dilation of Bowman's capsule, partial inosculation of foot processes of epithelial cell (Fig. 6A), proliferation of mesangial cell (Fig. 6B), and deformation of nuclei and hydropic mitochondria with pale matrices in proximal tubular cells (Fig. 6C), while in December, a normal subcellular structure of the kidney was observed, including foot processes in epithelial cell of glomeruli and elliptical nuclear and rich mitochondria in proximal tubular cells (Fig. 6D).

During the field study period, there were great temporal variations in the activities of antioxidant enzymes CAT, SOD, GPX, GST, and the concentration of GSH in the liver and kidney of bighead carp collected from the pen in Meiliang Bay (Fig. 7). From June to October, when toxic *Microcystis* blooms occurred, bighead carp showed higher activities of CAT, SOD, and GST, and lower concentrations of GSH than before or after *Microcystis* blooms in both liver and kidney. The GPX activity in the liver reached the maximum in June 2004 and then decreased progressively. In contrast, GPX in the kidney showed a complex fluctuation during the study period. Significant differences in activities of CAT and GST in the liver and kidney were observed before, during, and after *Microcystis* blooms (Table 3). On the other hand, the activity of the antioxidant enzymes was strongly tissue dependent; CAT, GST, and GSH except SOD and GPX had higher activities in liver than in kidney ($p < 0.001$ in CAT, $p < 0.05$ in GST, and $p < 0.01$ in GSH).

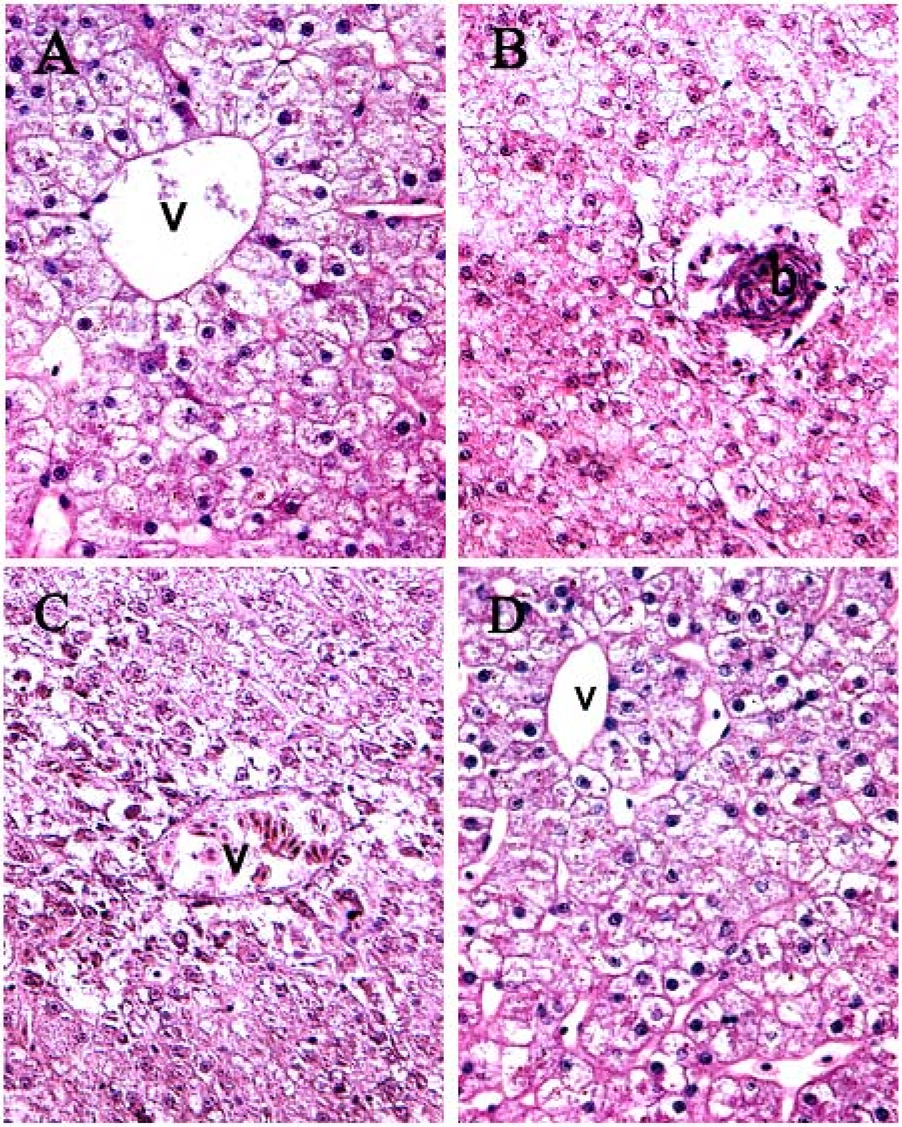

**FIGURE 3.** H&E-stained liver of sections of bighead carp collected from the fish pen in Meiliang Bay of Taihu Lake (400× magnification; v, central vein). (A) An example of a normal liver, which was the specimen obtained during April and May, 2004; (B and C) the examples of hepatocyte vacuolation and disorganization of cell structure in periportal and central region, which were typical of liver sections obtained during July and August, 2004; (D) an example of recovery of liver structure, which was typical of liver sections obtained from November 2004 to March 2005.

### DISCUSSION

In our laboratory study, when fish were injected i.p. with 200 and 500 µg MC-LReq/kg bw, respectively, liver pathology in bighead carp was observed in a time dose-dependent manner within 24 h postinjection and characterized by disruption of liver structure, condensed cytoplasm, and the appearance of massive hepatocytes with karyopyknosis, karyorrhexis, and karyolysis, which are comparable to previous studies in carnivorous rainbow trout[9,11,35] and in omnivorous common carp[7,12]. Unlike fish injected i.p. with MCs when histological lesions start from the centrilobular region[7,9], field fish exposed to MCs in the present study showed a temporal sequence of liver damage as follows: swelling/vacuolation of periportal hepatocytes, more severe vacuolation in midzonal and centrilobular hepatocytes. It seems that

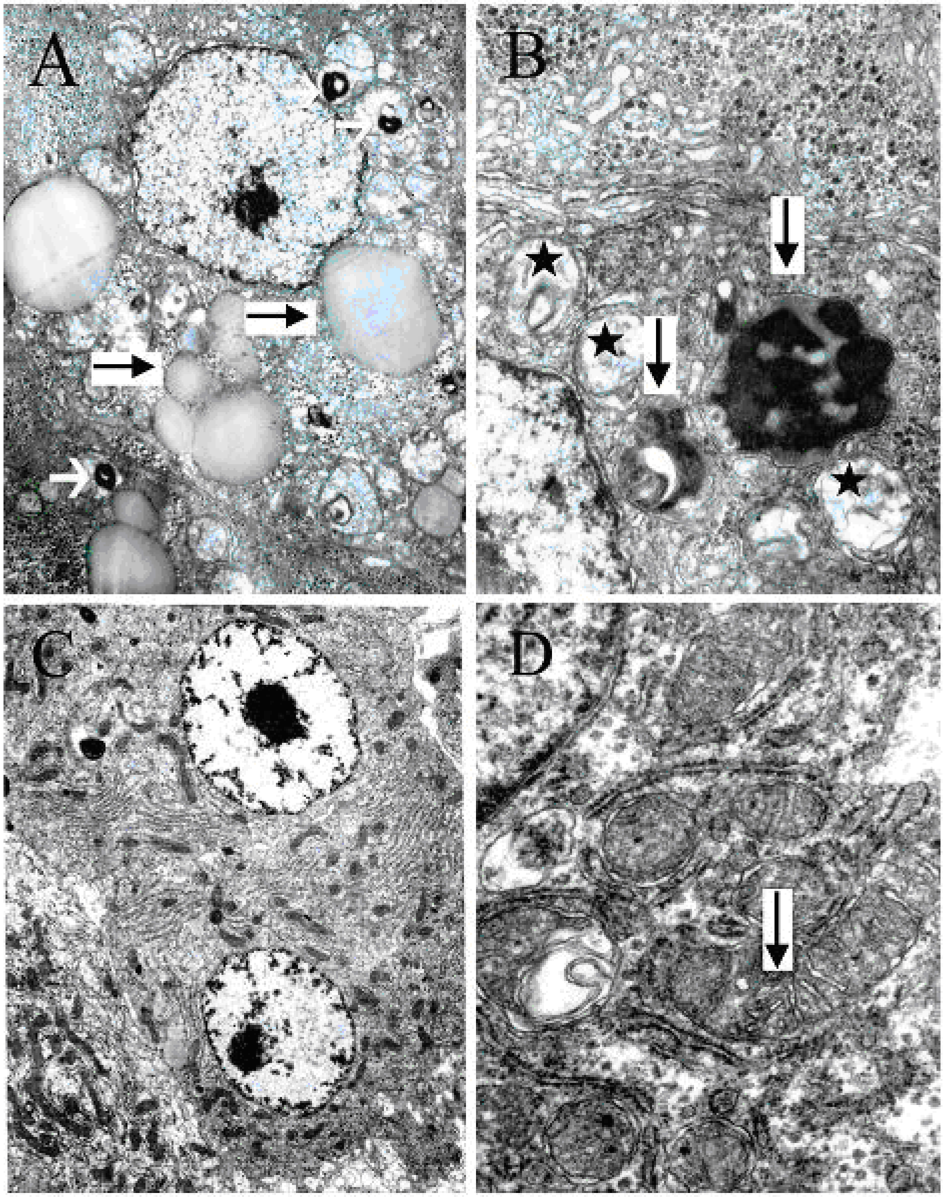

**FIGURE 4.** Transmission electron micrograph of the liver of bighead carp collected from the fish pen in Meiliang Bay of Taihu Lake during July (A and B) and December (C and D), 2004. (A) The increase of lipid droplets in size and number (black arrows) and the presence of vacuoles with myelin bodies (white arrows), 8000×; (B) augmentation of the amount of secondary lysosomes (black arrows) and some vacuoles (asterisks), 15000×; (C) the intact hepatocytes with abundant mitochondria and stacks of endoplasmic reticulum, 6000×; (D) normal mitochondria with clear cristae (black arrows), 20,000×.

different exposure routes may lead to different uptake pathways of MCs into the liver. Blood always flows from the periphery to the center of the liver lobule. Consequently, in the case of gavaging, MCs absorbed in the intestines first reach the peripheral cells via the bloodstream and then the central cells of the liver lobule, while through i.p. injection, the liver is directly exposed to MCs. This may partly explain why gavaging appears to be less harmful than i.p. injection. It is interesting to note that bighead carp in our field and laboratory studies underwent progressive histological recovery. To our knowledge, there is only one study to report recovery of tissue structure and regeneration of hepatocytes in goldfish *C. auratus* injected i.p. with a dose of 125 μg MC-LR/kg bw after 96 h, and there was a reconstruction of initial tissue

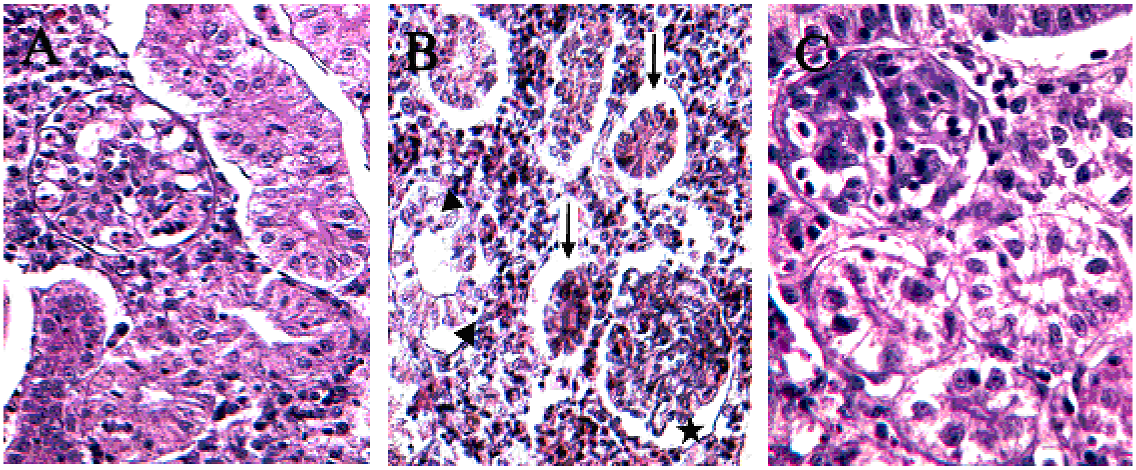

**FIGURE 5**. H&E-stained kidney sections of bighead carp collected from the fish pen in Meiliang Bay of Taihu Lake. (A) An example of a normal kidney, which was the specimen obtained during April and May, 2004, 400×; (B) an example of dilation of Bowman's capsule in the glomeruli (asterisk), vacuolation (arrows), and atrophy (arrow heads) in the proximal tubules, which was typical of kidney sections obtained during July and August, 2004, 400×; (C) an example of recovery of kidney structure, which was typical of liver sections obtained from November 2004 to March 2005, 600×.

structure concomitant with a decrease of plasma enzyme activity 21 days postinjection to values similar to those of the control fish[10]. Thus, it seems that compared with goldfish, bighead carp recovered more quickly in spite of exposure to higher MC doses. Such differences in response to MC exposure are most likely due to a different capability of detoxification of MCs among different fish species, since phytoplanktivorous fish like silver and bighead carp are possibly more tolerant to high MCs than other fish from an evolutionary point of view[25].

There are a few studies describing pathological changes of fish liver by oral uptake of MCs under laboratory conditions[8,11,13,36] and in natural contaminated waters[36]. In rainbow trout, damage of hepatocytes can be observed as early as 1 h after gavaging with freeze-dried toxic cells of *M. aeruginosa* at a single dose of 5700 μg MC-LReq/kg bw, and the alterations included a disassociation of hepatocytes, an early onset of apoptotic cell death, and delayed cell lysis[11,36]. In common carp gavaged with either the whole *M. aeruginosa* cells, lysed *M. aeruginosa* cells, or pure MCs at a dose of 2.5 μg/kg bw for 16 days, Carbis et al.[8] reported a loss of liver structural architecture and widespread necrosis of hepatocytes. When common carp were fed with bloom scum at a dose of 50 μg MC-LR/kg bw for 28 days, the ultrapathological examination displayed a widespread swelling and vacuolation of the hepatocytic endomembrane system (mainly consisting of endoplasmic reticulum, mitochondria, and Golgi body)[13]. In a similar field study, carp were exposed to *M. aeruginosa* at Lake Mokoan (Australia), where the total concentration of MCs was approximately 4.0 μg/g of the lyophilized scum material, and liver histology was characterized by cytoskeletal collapse, cytoplasmic vacuolation, pyknosis, eosinophilia, and widespread hepatocyte atrophy during February, March, and April[37]. In spite of different oral gavaging doses and modes, the severe injury in rainbow trout and common carp possibly caused hepatocytic necrosis[8,11,12,13]. In our field experiment, the daily dose of bighead carp was estimated to be 1358~4094 μg/kg bw during June to October, assuming an average daily ratio of 6.79%[38], and the concentration of MCs in cyanobacteria samples collected from the fish pen ranged from 200~603 μg/g dry algae (unpublished data of Dr. H. Yang). Apparently, in comparison to rainbow trout and common carp, bighead carp endured higher MC doses and longer-term exposure (not less than 5 months), but displayed slight histo- and ultrapathological changes. In our field study, lysosomal proliferation occurred in hepatocytes of bighead carp during *Microcystis* blooms, which agrees with previous *in vitro* studies on fish cell lines (RTG-2 and PHLC-1), where there was a very potent concentration-dependent stimulation of the lysosomal function by MC-LR[39], probably related to

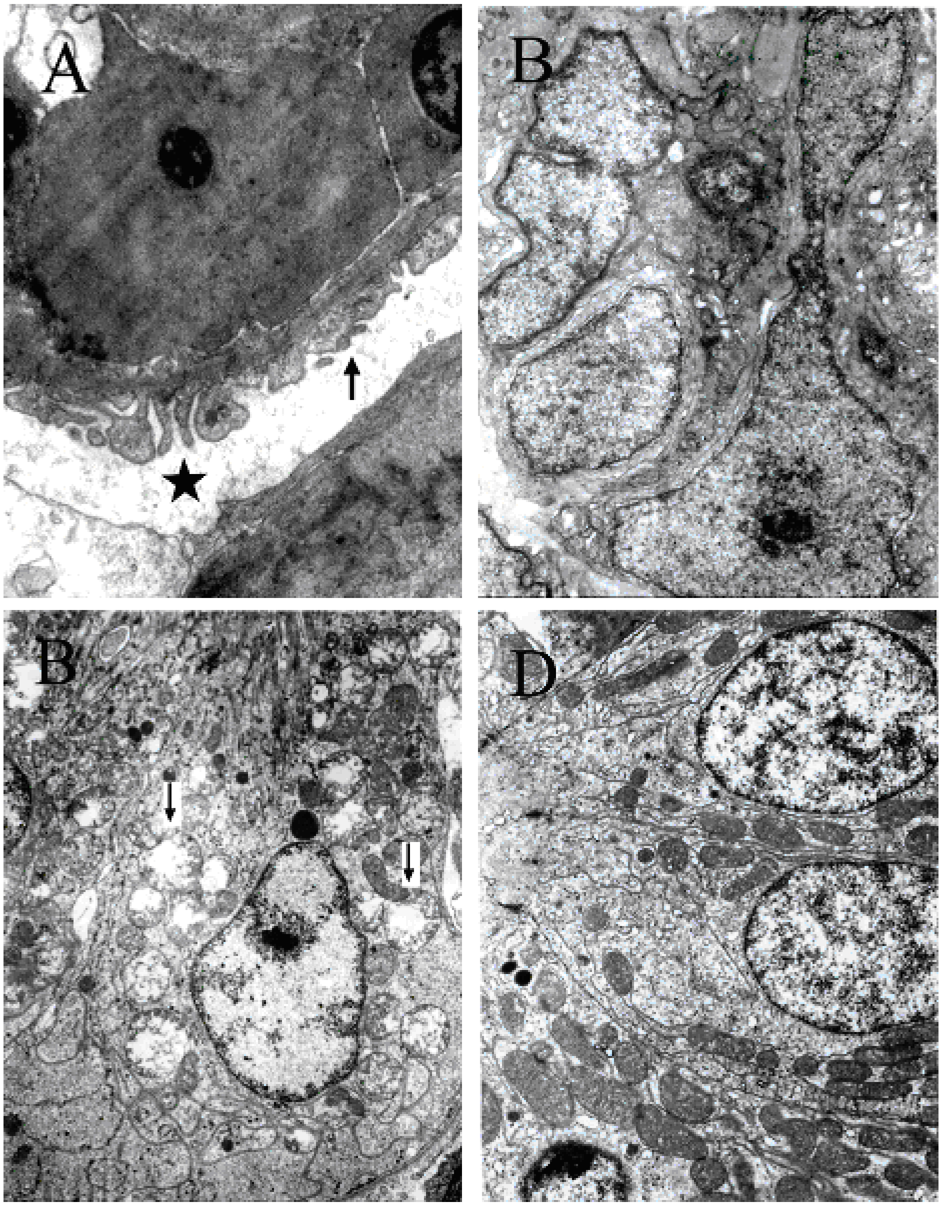

**FIGURE 6.** Transmission electron micrograph of the kidney of bighead carp collected from the fish pen in Meiliang Bay of Taihu Lake during July (A and C) and December (D and E), 2004. (A) Dilation of Bowman's capsule in glomeruli (asterisk) and inosculation of foot processes in epithelial cell of glomeruli (arrows), 10,000×; (B) proliferation of mesangial cells in glomeruli, 10,000×; (C) deformation of nuclei and hydropic mitochondria with pale matrices in proximal tubular cells, 6000×; (D) normal proximal tubular cells with elliptical nuclear and rich mitochondria, 6000×.

cytoskeletal modifications and the induction of oxidative stress[40,41]. Braunbeck[42] reported proliferation of lysosomes in the hepatocytes of rainbow trout following *in vivo* and *in vitro* sublethal exposure to xenobiotics (e.g., 4-chloroaniline), which indicates a general adaptation to compensate for increased turnover of cellular components under conditions of toxicant-induced stress. Therefore, the proliferation of lysosomes in our study may indicate an adaptive mechanism of bighead carp to eliminate or lessen cell damage caused by MCs through lysosome activation.

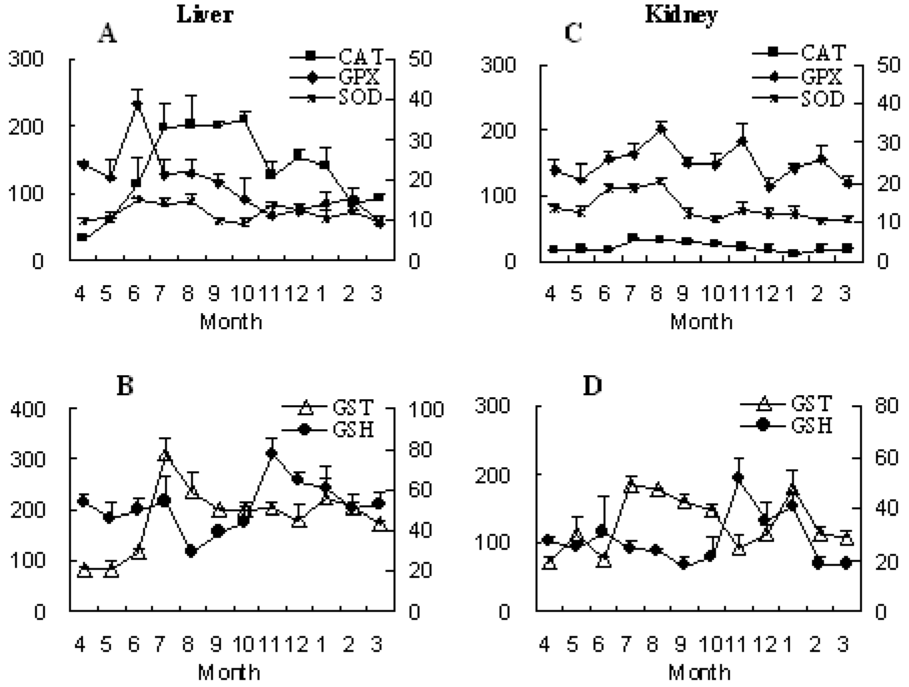

**FIGURE 7.** Seasonal variations of the activities of CAT (µmoles/min/mg protein), SOD (U/mg protein), and GPX (nmoles/min/mg protein), and GST (nmoles/min/mg protein), and GSH concentrations (µg/mg protein) in liver (A and B) and kidney (C and D) of bighead carp collected from the fish pen in Meiliang Bay of Taihu Lake during April 2004 and March 2005. Results was expressed mean ± SD (n = 5).

During our field study, kidney lesions in bighead carp were indicated by dilation of Bowman's capsule, partial inosculation of foot processes of epithelial cell and proliferation of mesangial cells in glomeruli, and hydropic mitochondria in proximal tubules, which are to some extent consistent with those obtained from carp that were immersed in tanks containing MCs from cyanobacterial cells[8] or gavaged with a single bolus dose of MC-LR[12]. After being absorbed via the intestinal cells, MCs are transported to various organs or tissues, especially liver, kidney, gills, etc., which are highly blood irrigated[43]. An immunostaining study found that the injected cysteine conjugate of MC-LR (MCLR-Cys) was the most actively excreted from the kidney, indicating that the kidney is also involved in eliminating MC-LR from the body[44]. Thus, it is not surprising to find pathological changes in the kidney of bighead carp during our field study. Indeed, a rapid and high accumulation of MCs (0.029~1.696 µg/kg DW) in the kidney was observed in bighead carp collected from the fish pen[45], suggesting the possibility of chronic nephrotoxicosis.

Antioxidant components and antioxidant enzymes, which are generally ubiquitous in aquatic organisms, counteract endogenous and exogenous oxidative stress and play an important role in decomposing some toxic xenobiotics. Both in field and laboratory studies, a reversible variation in the activities of the antioxidant enzymes CAT, SOD, GPX, and GST was observed, along with quick structural recovery of the liver and kidney. The significant increases in the antioxidant enzymes indirectly verified that oxidative stress occurred in bighead carp. It appears that MC-induced oxidative stress activated the antioxidant enzymes, which probably cooperated to eliminate ROS so as to protect cells from lesions. Similar results are also observed in the liver of loach (*Misgurnus mizolepis*) that were orally exposed to a low dose of *Microcystis* (equal to 10 µg MC-RR/kg bw) through dietary supplementation with bloom scum and in liver, kidney, and gill tissues of tilapia (*Oreochromis* sp.) exposed during 21 days to cyanobacterial cells mixed with their food[16,46]. On the other hand, GSH is involved in a number of crucial cellular functions, such as ROS scavenging, detoxification of xenobiotics, maintenance

**TABLE 3**
**The Activities of Antioxidant Enzymes CAT, SOD, GPX, and GST and GSH Levels in the Liver and Kidney of Bighead Carp Collected from the Fish Pen in Meiliang Bay of Taihu Lake During Three Periods (Before, During, and After *Microcystis* Blooms)**

| | | CAT | SOD | GPX | GST | GSH |
|---|---|---|---|---|---|---|
| Liver | Before *Microcystis* blooms (2004.4-5) | 45.6±19.6 | 10.2±0.4 | 133.1±13.6 | 83.7±1.6 | 49.3±5.4 |
| | During *Microcystis* blooms (2004.6-10) | **184.0±40.3*** | 12.7±2.9 | 140.3±53.8 | **213.6±74.8*** | 43.6±9.8 |
| | After *Microcystis* blooms (2004.11-2005.3) | 119.8±30.9 | 11.7±1.6 | 75.8±14.7 | 197.0±20.7 | 61.2±10.7 |
| kidney | Before *Microcystis* blooms (2004.4-5) | 17.1±2.1 | 11.6±1.4 | 132.3±12.1 | 92.1±29.6 | 25.9±1.8 |
| | During *Microcystis* blooms (2004.6-10) | **26.7±6.6*** | 14.0±4.1 | 163.8±22.7 | 149.0±43.7 | 23.4±5.0 |
| | After *Microcystis* blooms (2004.11-2005.3) | 16.7±3.6 | 12.4±3.3 | 142.5±28.7 | 121.2±33.2 | 32.7±14.5 |
| ANOVA Effects | | (B)P<0.001 (T)P<0.001 B*T P<0.01 | (B) N.S (T) N.S B*T N.S. | (B) P<0.05 (T) N.S B*T N.S. | (B) P<0.05 (T) P<0.05 B*T P<0.05 | (B)P<0.05 (T)P<0.01 B*T N.S. |

*Note:* Results are expressed as mean ± SD. Number of replicates for each group was 25, except for the period before *Microcystis* blooms (n = 10). The statistical significance was analyzed by two-way ANOVA with blooms (B) and tissue (T) as factors. Posthoc comparison for the effect of *Microcystis* blooms: Bonferroni's test, $p < 0.05$.

of thiol-disulfide status, and signal transduction[47]. Detoxification of MCs in livers is known to occur via their conjugation to GSH, a reaction catalyzed by GST[48,49]. This first step of detoxification has been described *in vivo* in mammals[50] as well as in aquatic plants, crustaceans, mollusks, and fish[48]. Augmented GST activity has been utilized to point out MC detoxification in various aquatic organisms ranging from microalgae, plants, invertebrates, to fish[19,51,52]. In this respect, hepatic GST activity as well as GSH concentration should be privileged biomarkers of exposure to MCs. Thus, the enhanced GST activities in our laboratory study and during the period of *Microcystis* blooms in our field study could be due to enhanced biotransformation reactions and higher rates of MC conjugation, suggesting the importance of MC detoxification by the GSH pathway. No obvious depletion in GSH levels was observed in bighead carp in field and laboratory studies, despite that the conjugation of MCs with GSH in the detoxification process is expected to induce a leak of this tripeptide from the intracellular pool.

The content of intracellular GSH is a function of the balance between its use and synthesis, and cellular responses to toxins and xenobiotics often involve changes in GSH content. Initially, GSH is consumed by conjugation via GST or by glutathione disulfide formation when GSH reacts with ROS. It is then replaced either by enzymatic reduction catalyzed by GR or by *de novo* synthesis. In primary cultured rat hepatocytes exposed to cyanobacteria extract, Ding et al.[53] suggested that the use of GSH by the initial conjugation to MC-LR may trigger an increase in GSH synthesis, possibly by activating the transcription of γ-glutamylcysteine synthetase, the rate limiting enzyme in GSH synthesis. Nevertheless, response of intracellular GSH content to MC exposure in fish has been quite different so far, probably due to different exposure dose, time, and routes, or different composition of MCs or different physiological characteristics of the selected tissue. Xu et al.[54] observed a decrease in GSH concentration in the liver of grass carp following an i.p. injection of MC-LR (10 μg MC-LR/kg and higher). A similar decrease was also demonstrated by Li et al.[6] on cultured hepatocytes of common carp exposed to MC-LR at a dose of 10 μg/l for 2, 4, and 6 h, respectively. This reduction of GSH content after MC-LR exposure may be explained by its increased utilization in detoxification processed, by a reduced GSH synthesis, or by both mechanisms. On the contrary, a significant increase in GSH level was reported in the liver of silver carp (*Hypophthalmichthys molitrix*) exposed to a natural population of cyanobacterial bloom for 25 days, indicating an increased GSH demand as a result of oxidative stress and/or induction of detoxification enzymes in MC-exposed fish[55]. Decrease in GSH content in the liver of juvenile goldfish was not significant after an i.p. injection of MC-LR (125 μg/kg) for 96 h[10], which was attributed to its important basic GSH concentration. In our study, the basic level of GSH in the liver of bighead carp was 57.8 ± 5.2 μg/mg protein, nearly 2.5 times that in the liver of *C. auratus* (22.16 ± 1.60 μg/mg protein)[56] and that in *Culter ilishaeformis* (23.2 μg/mg protein) in Lake Taihu (Qiu T, unpublished data), suggesting that the detoxification of MCs by the GSH pathway might not have caused a substantial decline in GSH level. Furthermore, GSH depletion or formation limits the ability of an organism to detoxify MCs or prevent oxidative damage[57]. Previous studies on toxicity of MC-LR on animal hepatocytic antioxidant systems also directly demonstrate that antioxidant systems, mainly GSH, could be relevant indices in explaining the sensitivity of some vertebral species to MCs[58]. So the stable GSH levels in bighead carp liver and kidney in both field and laboratory studies may be explained by the high basic GSH concentration in bighead carp liver and/or an increased GSH synthesis, suggesting a high ability to detoxify MCs and to release associated high-oxidative pressure in phytoplanktivorous fish. All of the above results provide physiological and toxicological evidence underlining the higher resistance of phytoplanktivorous fish to MC exposure and suggest that bighead carp can be used as a biomanipulation fish to counteract cyanotoxin contamination.

### ACKNOWLEDGMENTS

We would like to express our sincere thanks to Dr. Karl E. Havens, Dr. Micheal S. Allen, and the anonymous reviewers for their useful comments and suggestions on this manuscript. This work was supported by a fund from the National Natural Science Foundation of China (30530170) and by the Key Project of CAS titled “The effects of the regenerative organic pollutant microcystins on the safety of aquatic food” (Grant No. KSCX2-SW-129).
